# Effect of Small and Large Energy Surpluses on Strength, Muscle, and Skinfold Thickness in Resistance-Trained Individuals: A Parallel Groups Design

**DOI:** 10.1186/s40798-023-00651-y

**Published:** 2023-11-02

**Authors:** Eric R. Helms, Alyssa-Joy Spence, Colby Sousa, James Kreiger, Steve Taylor, Dustin J. Oranchuk, Brad P. Dieter, Casey M. Watkins

**Affiliations:** 1 Sport Performance Research Institute New Zealand (SPRINZ), Auckland University of Technology, 17 Antares Place, Rosedale, Auckland, 0632 New Zealand; 2 Muscle Physiology Laboratory, Department of Exercise Science and Health Promotion, Florida Atlantic University, Boca Raton, FL USA; 3 Weightology, LLC, Issaquah, WA USA; 4 Steve Taylor RD, Kansas City, MO USA; 5 Macros Inc, Las Vegas, NV USA; 6 Department of Kinesiology, Seattle University, Seattle, WA USA

**Keywords:** Resistance training, Body composition, Hypertrophy, Energy surplus

## Abstract

**Background:**

Many perform resistance training (RT) to increase muscle mass and strength. Energy surpluses are advised to support such gains; however, if too large, could cause unnecessary fat gain. We randomized 21 trained lifters performing RT 3 d/wk for eight weeks into maintenance energy (MAIN), moderate (5% [MOD]), and high (15% [HIGH]) energy surplus groups to determine if skinfold thicknesses (ST), squat and bench one-repetition maximum (1-RM), or biceps brachii, triceps brachii, or quadriceps muscle thicknesses (MT) differed by group. COVID-19 reduced our sample, leaving 17 completers. Thus, in addition to Bayesian ANCOVA comparisons, we analyzed changes in body mass (BM) with ST, 1-RM, and MT changes via regression. We reported Bayes factors (BF_10_) indicating odds ratios of the relative likelihood of hypotheses (e.g., BF_10_ = 2 indicates the hypothesis is twice as likely as another) and coefficients of determination (*R*^2^) for regressions.

**Results:**

ANCOVAs provided no evidence supporting the group model for MT or squat 1-RM. However, moderate (BF_10_ = 9.9) and strong evidence (BF_10_ = 14.5) indicated HIGH increased bench 1-RM more than MOD and MAIN, respectively. Further, there was moderate evidence (BF_10_ = 4.2) HIGH increased ST more than MAIN and weak evidence (BF_10_ = 2.4) MOD increased ST more than MAIN. Regression provided strong evidence that BM change predicts ST change (BF_10_ = 14.3, *R*^2^ = 0.49) and weak evidence predicting biceps brachii MT change (BF_10_ = 1.4, *R*^2^ = 0.24).

**Conclusions:**

While some group-based differences were found, our larger *N* regression provides the most generalizable evidence. Therefore, we conclude faster rates of BM gain (and by proxy larger surpluses) primarily increase rates of fat gain rather than augmenting 1-RM or MT. However, biceps brachii, the muscle which received the greatest stimulus in this study, may have been positively impacted by greater BM gain, albeit slightly. Our findings are limited to the confines of this study, where a group of lifters with mixed training experience performed moderate volumes 3 d/wk for 8 weeks. Thus, future work is needed to evaluate the relationship between BM gains, increases in ST and RT adaptations in other contexts.

## Key Points

- When assigning intended energy surplus sizes of 5–15%, faster rates of body mass gain primarily serve to increase the rate that fat mass accumulates, rather than increasing rates of hypertrophy or strength gain.
- It is possible, however, that faster rates of body mass gain could enhance hypertrophy to some degree if a sufficient training stimulus is provided.
- While further work is needed, if a sufficient training stimulus is provided, the rate of body mass gain that will best support hypertrophy is likely individual. Rates may be influenced by prior training experience, hereditary factors which influence one’s potential maximum rate of muscle gain, and other variables (sleep, stress, etc.).

## Background

Resistance training (RT) is widely used in strength and conditioning, fitness, and clinical settings to elicit muscular adaptations, including skeletal muscle hypertrophy. More specific to those who regularly and primarily perform progressive RT, in strength sports such as powerlifting, lean mass is strongly related to competitive success [1]. Additionally, in competitive bodybuilding, a more muscular physique contributes largely to one’s score [2]. Therefore, athletes, personal trainers, strength and conditioning coaches, and sports nutrition professionals can all benefit from a better understanding of how to increase muscular hypertrophy.

While RT provides the initial stimulus that can result in changes in muscle mass, other factors can influence the magnitude of these changes. Specifically, research over the last several decades has demonstrated that nutritional factors can have substantial effects on muscle mass accrual [3–6]. Multiple aspects of nutrition can influence muscular hypertrophy. For example, consuming a sufficiently high daily protein intake can augment gains in lean mass in response to RT [7–9]. Further, trainees may be able to perform greater volumes of RT, which are associated with greater hypertrophy [10] when consuming sufficient carbohydrate before training [11]. More broadly, an energy surplus could theoretically aid hypertrophy considering it is an energetically expensive process, that RT itself increases energy expenditure, and since energy expenditure can increase in response to overfeeding [6]. Conversely, sufficiently large energy deficits are associated with blunted hypertrophy [12]. Further, insufficient intakes of carbohydrate [13] or fat [14]—which are more likely when consuming insufficient energy—may result in hormonal environments potentially less conducive for hypertrophy.

While these findings may prompt a recommendation to practice overfeeding to optimize hypertrophy, doing so may not be required as increases in muscle mass have been observed, while fat loss occurs [15]. Therefore, while not a prerequisite, an energy surplus (or at least the absence of a deficit) may support hypertrophy. What is not yet clear, is whether a larger surplus is more effective than a smaller surplus for supporting hypertrophy, or if simply performing RT while at energy balance is sufficient. Indeed, when different magnitudes of overfeeding are experimentally induced, hypertrophy is inconsistently enhanced, with surpluses that are too large sometimes resulting in increases in fat mass without additional muscle gain beyond that provided by RT alone [16, 17]. Thus, while prescribing a large surplus may ensure RT adaptation is supported, too large of a surplus may result in unwanted fat mass gain, potentially prompting a subsequent fat loss phase depending on individual goals. An important next step in applied research is to determine the optimal surplus size which maximizes muscle gain while minimizing fat gain.

Therefore, the purpose of this study was to compare RT-induced hypertrophy and strength gains in three groups of resistance-trained participants after eight weeks of supervised training while consuming three different energy intakes: (1) an estimated 15% surplus (HIGH), (2) an estimated 5% surplus (MOD), and (3) estimated maintenance (MAIN). These surplus sizes were chosen based on the findings of Garthe and colleagues [17], who reported non-significant strength and lean body mass differences in two groups of athletes consuming similarly sized surpluses to those used in this study, but also significantly higher fat mass gain in their higher surplus group. Further, the 15% surplus is roughly in the middle of Iraki and colleagues' [3] surplus size recommendations for off-season competitive natural bodybuilders, while 5% is at the lower end, representing a “standard” approach to muscle gain and an approach attempting to minimize fat gain, respectively. We hypothesized that both surplus groups would increase muscle size and strength to a greater degree than the MAIN but would make similar gains to one another. Further, we hypothesized that increases in body fat would be directly related to the size of the energy surplus.

## Methods

### Participants

Twenty-one trained individuals (2 females, 19 males; Table 1) participated in this study. Four male participants were unable to complete the study protocol and were therefore not included in the analysis. Therefore, the final sample size was 17. Reasons for discontinuation included COVID-19 regulations and sickness. One participant experienced pain during squat post-testing which prevented completion of the post-test squat 1RM, all other data for this participant were used. Data were included only if participants completed pre- and post-testing; all participants completed > 90% of training sessions. For inclusion, participants were required to have at least one year of RT experience, defined as performing RT sessions at least twice weekly including the bench press and squat exercises each at least once per week with no complete breaks from training longer than two weeks in succession. Further, participants needed a minimum strength level of a 1 × or 0.75 × bodyweight bench press and a 1.5 × or 1.25 × bodyweight squat for men and women, respectively. These criteria, as well as the ability to perform the back squat and bench press required technique, were confirmed during the familiarization session. All participants were injury-free at the time of inclusion. Participants using anabolic steroids or other anabolic performance-enhancing drugs were excluded from the study. Moreover, all participants were informed of the risks and benefits associated with this study, after which they signed an institutionally approved written consent prior to data collection. The methods and procedures used in this study were approved by the first author’s University ethics board (Approval Number 18/53).

**Table 1:** Participant characteristics (mean ± SD)

	All (*n* = 17)			MOD (*n* = 6)			HIGH (*n* = 5)			MAIN (*n* = 6)		
	Pre	Post	Change	Pre	Post	Change	Pre	Post	Change	Pre	Post	Change
Age (y)	27.2 ± 3.8			28.8 ± 2.6			28.6 ± 3.4			24.3 ± 3.9		
Height (cm)	173.2 ± 7.5			174.0 ± 7.2			174.6 ± 6.2			171.3 ± 9.5		
Body mass (kg)	77.5 ± 11.7	79.8 ± 12.1	2.3 ± 1.7	79.2 ± 7.2	82.5 ± 7.3	3.3 ± 0.8	82.6 ± 7.5	85.9 ± 6.9	3.3 ± 1.5	71.7 ± 16.3	72.1 ± 16.1	0.4 ± 0.5
Bench 1RM (kg)	98.4 ± 22.8	106.9 ± 23.9	8.5 ± 4.4	100.6 ± 29.3	107.9 ± 31.6	7.3 ± 3.3	103.6 ± 11.6	117.0 ± 11.1	13.4 ± 1.5	91.8 ± 24.8	97.5 ± 22.9	5.7 ± 3.9
Relative Bench (1RM/bm)	1.27 ± 0.21	1.34 ± 0.22	0.07 ± 0.05	1.26 ± 0.33	1.30 ± 0.35	0.04 ± 0.04	1.26 ± 0.11	1.36 ± 0.09	0.11 ± 0.02	1.28 ± 0.15	1.36 ± 0.16	0.08 ± 0.06
Squat 1RM (kg)	139.7 ± 33.9*	150.4 ± 33.4*	10.7 ± 6.8	137.0 ± 44.5*	148.6 ± 44.5*	11.6 ± 7.2	147.3 ± 25.6	158.0 ± 27.1	10.7 ± 27.1	135.7 ± 35.4	145.7 ± 33.0	10.0 ± 7.7
Relative Squat 1RM (1RM/bm)	1.80 ± 0.34*	1.89 ± 0.33*	0.09 ± 0.09	1.72 ± 0.52*	1.80 ± 0.50*	0.08 ± 0.08	1.78 ± 0.19	1.83 ± 0.22	0.06 ± 0.06	1.89 ± 0.29	2.02 ± 0.22	0.13 ± 0.12
Sum of 8 SF (cm)	80.9 ± 27.8	87.6 ± 31.9	6.7 ± 9.8	90.3 ± 28.1	100.2 ± 32.2	10.0 ± 9.0	77.6 ± 27.8	90.1 ± 28.8	12.4 ± 8.3	74.4 ± 29.9	73.0 ± 33.3	-1.4 ± 6.8
VL MT (cm)	2.61 ± 0.44	2.71 ± 0.36	0.10 ± 0.20	2.81 ± 0.55	2.95 ± 0.46	0.14 ± 0.16	2.43 ± 0.23	2.54 ± 0.17	0.11 ± 0.21	2.57 ± 0.43	2.62 ± 0.27	0.05 ± 0.24
Lat VI MT (cm)	2.33 ± 0.39	2.34 ± 0.32	0.01 ± 0.21	2.36 ± 0.25	2.38 ± 0.24	0.02 ± 0.13	2.52 ± 0.45	2.48 ± 0.45	-0.04 ± 0.19	2.15 ± 0.44	2.19 ± 0.24	0.03 ± 0.30
Ant VI MT	2.66 ± 0.62	2.87 ± 0.56	0.22 ± 0.42	2.36 ± 0.39	2.62 ± 0.59	0.26 ± 0.28	2.88 ± 0.80	2.99 ± 0.70	0.11 ± 0.35	2.77 ± 0.61	3.03 ± 0.37	0.26 ± 0.62
RF MT	2.63 ± 0.49	2.54 ± 0.47	-0.09 ± 0.39	2.61 ± 0.36	2.76 ± 0.43	0.15 ± 0.16	2.73 ± 0.83	2.52 ± 0.58	-0.21 ± 0.26	2.56 ± 0.28	2.34 ± 0.38	-0.22 ± 0.56
TB MT	4.71 ± 0.85	4.68 ± 0.90	-0.03 ± 0.47	5.05 ± 1.09	4.80 ± 1.14	-0.25 ± 0.57	4.79 ± 0.64	4.90 ± 0.66	0.11 ± 0.37	4.30 ± 0.68	4.38 ± 0.88	0.08 ± 0.39
BB MT	3.11 ± 0.57**	3.37 ± 0.55**	0.26 ± 0.36	3.15 ± 0.65*	3.35 ± 0.55*	0.19 ± 0.10	3.44 ± 0.37	3.79 ± 0.55	0.34 ± 0.54	2.73 ± 0.50*	2.98 ± 0.21*	0.25 ± 0.37

*n = − 1, **n = − 2; see “Participants” section, one participant was unable to complete squat 1RM post-test, two participants did not have BB MT measurements

*1RM* one-repetition maximum, *bm* body mass, *SF* skin folds, *lat* lateral, *VL* vastus lateralis, *MT* muscle thickness, *ant* anterior, *VI* vastus intermedius, *RF* rectus femoris, *TB* triceps brachii, *BB* biceps brachii

### Procedures

Participants visited the laboratory 25 times over eight weeks, these visits included pre- and post-testing, and each supervised training session. Nutrition intake was monitored throughout. All groups performed a thrice-weekly, full-body, hypertrophy-oriented supervised RT program. Group allocation was blinded for researchers supervising training sessions to mitigate any bias surrounding testing and training. Pre- and post-testing included a calliper skinfold assessment, an ultrasound assessment of upper and lower body muscle thickness (MT), and a one-repetition maximum (1-RM) test for bench press and back squat exercises. Testing sessions were completed 48–72 h before the first training session and 48–72 h following the last training session.

### Pre-intervention Maintenance and Familiarization Phase

Before the intervention, all participants underwent a maintenance and familiarization phase. The goals of this phase were for participants to establish the habit of weighing themselves every morning, tracking their food intake in a food tracking app in real time (i.e., as they ate throughout the day), and to achieve weight stability.

Before beginning the maintenance and familiarization phase, participants received nutrition guidance from one of two research team members with concentrated experience regularly working with remote clients seeking to alter their body composition. Of the two nutrition research team members, one is a registered dietitian and the other an experienced clinical nutrition researcher with a PhD in exercise physiology. These research team members monitored nutrition intake via an online food tracking software (MyFitnessPal, California, USA) and conducted weekly check-ins. Initially, all participants met via video call with one of the two nutrition research team members. During this call, participants were instructed how to weigh themselves (e.g., first thing in the morning, after going to the bathroom, in a fasted state, and nude), given a detailed video tutorial demonstrating the proper way to track macronutrients, as well as instructions for completing their weekly check-ins. Participants remained in the maintenance and familiarization phase until they achieved weight stability. Weight-stable was defined as having the same weekly average body weight (± 1%) for two successive weeks. During this phase the participants maintained their habitual training.

### Nutrition Interventions

Once a participant was determined weight-stable, they were randomly assigned to a group using alternating pattern of MAIN, MOD, HIGH upon completion of the maintenance phase and enrolment in the full protocol. Only the nutrition supervising researcher was aware of group assignment, while the researchers supervising training were blinded to group assignment. Further, participants were asked to keep their group assignment to themselves when interacting with the researchers supervising training. Groups differed by their energy intake, with MAIN assigned an energy intake target predicted to keep their initial weight stable, within ± 1%, as defined during the maintenance phase. The MOD was assigned an energy intake target predicted to increase body weight by 0.4–0.6% every two weeks. Finally, HIGH was assigned an energy intake target predicted to increase body weight by 1.4–1.6% every two weeks.

All groups were given the same instructions regarding their macronutrient intake. Instructions required participants to consume a minimum of 1.8 g of protein per kilogram of body weight, a minimum of 20% of energy from fat, and a minimum of 40% of energy from carbohydrate sources. Macronutrient and energy intakes among groups during the maintenance and familiarization phase and intervention are shown in Table 2. Outside of these constraints, participants could modify their macronutrient distributions to individual preferences. Further, all participants were instructed to consume their food during three to five meals spread relatively evenly throughout the day, while ingesting at least 20 g of protein within two hours of finishing their training sessions. Participants were instructed to maintain their supplement usage for the duration of the study and not to change supplement strategy, or doses consumed during the maintenance and familiarization phases.

**Table 2:** Macronutrient breakdown by group as a percentage of energy intake (mean ± SD)

	Protein		Fat		Carbohydrates		Kilocalories		
	Pre	Inter	Pre	Inter	Pre	Inter	Pre	Inter	Change
MAIN	25 ± 7	23 ± 4	31 ± 5	30 ± 4	44 ± 10	46 ± 1	2358 ± 661	2527 ± 647	169 ± 205
HIGH	26 ± 6	22 ± 2	34 ± 6	32 ± 3	40 ± 8	46 ± 3	2535 ± 265	3253 ± 262	719 ± 189
MOD	25 ± 4	22 ± 2	32 ± 6	30 ± 3	43 ± 8	48 ± 4	2645 ± 224	3135 ± 333	489 ± 184

*Pre* maintenance period, *Inter* intervention period

### Weekly Nutrition Check-Ins and Adjustments

Once per week, each participant would check in with their nutrition supervising researcher. For each check-in, participants were instructed to verify accuracy for their spreadsheet where they recorded their weight, energy, protein, fat, and carbohydrate intake each day. Additionally, participants were directed to send a video report (or occasionally an email when unable to record a video) in which they would summarize their week and ask any pertinent questions regarding the nutrition intervention. Common questions were typically related to how to track various foods as accurately as possible and requests for advice on how to achieve energy and macronutrient targets.

Upon review, if a participant’s body weight aligned with the desired rate of weight gain (or lack thereof) for their assigned group, no nutritional adjustments were made. However, if a participant’s body weight did not align with the desired rate of weight gain for their group (i.e., fell below or above the target rate of weight gain, or weight stability) adjustments to the assigned energy intake (and thus macronutrients) were made based on estimated relationships between weight gain, loss, and energy intake target [18]. The nutrition supervising researcher responded to participants via video within 24 h of receiving weekly information, answering questions, providing guidance, and detailing instructions for of any changes to their energy or macronutrient goals.

### Ultrasound Collection and Analysis

For pre- and post-testing, each participant’s height and body mass were taken upon arrival. Thereafter, participants lay supine on a massage table with their knees and hips fully extended for 10 min, to allow for inter- and intra-cellular fluid re-distribution [19]. The length of the lateral aspect of the thigh was measured as the distance from the superior border of the greater trochanter to the inferior border of the lateral condyle of the femur. The anterior aspect of the thigh was measured as the distance between the superior border of the patella and the inferior border of the anterior, superior iliac spine. Thigh lengths were recorded and marked with an indelible pen, 50% distance between the lateral and anterior borders. The vastus lateralis (VL) and lateral vastus intermedius (VI) were collected in one image, and the rectus femoris (RF) and anterior vastus intermedius were collected in one image [20, 21]. The vastus medialis was excluded as it can be further broken down into the obliquus and longus portions, with deep and superficial fiber bundles making consistent collection challenging [22].

In vivo muscle architecture was determined via 2-dimensional B-mode ultrasonography using an ultrasound transducer and built-in software (45 mm linear array, 10 MHz; GE Healthcare, Vivid S5, Chicago, IL, USA). On each occasion, two images were be captured and averaged to provide mean MT. A water-soluble gel was applied to the scanning head of the ultrasound probe to achieve acoustic coupling, with care taken to avoid the deformation of muscle architecture [23]. The transducer was positioned in the longitudinal plane to increase ease and reduce the time required to collect ultrasound images [24], and transducer tilting was carefully avoided [25]. The lateral thigh images were collected before the anterior thigh. Ultrasound settings (frequency: 12 MHz, brightness: maximum, gain: 60 dB, dynamic range: 70) were kept consistent across all participants. Due to large differences in MT, scanning depth was individualized for each participant and muscle, whereby settings were recorded and maintained through all collections [20, 21, 23]. Immediately following the lower body ultrasound collection, the participant stood up and the anthropometrist marked 50% of the length of the humerus as determined by the halfway point between the olecranon process and the lateral border of the acromion process. The center of the biceps and triceps brachii was marked with an indelible pen. Biceps and triceps images were collected in the transverse plane as no reliability or variability data exist for longitudinally collected images. All ultrasound settings and practices from the lower body assessment were applied to the biceps and triceps brachii. Intrasession variability of mid-region VL, RF, and lateral VI and anterior VI MT (ICC = 0.93–0.98, CV = 2.4–5.7%, TEM = 0.15–0.25) of resistance-trained men was previously determined in our laboratory [20, 21]. Additionally, Jenkins et al. [26] and Radaelli et al. [27] reported similar test–retest statistics for biceps brachii MT (ICC = 0.91–0.99, CV = 4.2%) in untrained men and women, respectively.

Images were analyzed via digitizing software (ImageJ; National Institutes of Health, USA). MT (cm) was defined as the perpendicular distance between the deep and superficial aponeurosis, and the deep aponeurosis. All images were inspected and analyzed by the same experienced sonographer [20, 21].

### Skinfold Assessment

Once all ultrasound imaging protocols were completed, body composition assessments were completed via an ISAK certified level 1 anthropometrist and included the standardized eight-site skinfold profile and associated girth assessments. Skinfold assessments were measured with Harpenden calipers (Baty International, England, UK) to the nearest 0.1 mm wherein measurement calibration was confirmed pre-trial. All anatomical reference points were measured according to standardized ISAK protocols, followed by skinfold assessments for triceps, subscapular, biceps, iliac crest, supraspinale, abdominal, front thigh, and medial calf regions. The sum of all eight sites was used in analysis. This ISAK skinfold profile was performed and repeated to verify measurement accuracy and mitigate any fluctuations in body fat pliability. A third measurement was taken for any variable which resulted in a difference in measurement scores greater than 5% or 1 mm, whichever value was less.

### One-Repetition Maximum Testing

Participants performed a standardized dynamic warm-up, followed by a specific warm-up based on individual 1-RM estimations. The specific warm-up included one set of 10 repetitions with the barbell, followed by one set each of 5 repetitions with 50%, 4 repetitions with 60%, 3 repetitions with 70%, 2 repetitions with 80%, and 1 repetition with 90% of estimated 1-RM load. Rest periods were three to five minutes long and given after each warm-up set and 1-RM attempt, starting after 70%. Participant repetition in reserve-based rating of perceived exertion (RPE) score [28] and their average concentric velocity (PowerTool, GymAware, Kinetic Performance Technology, Canberra, Australia) were used to direct 1-RM attempt selection. A 1-RM was recorded if the participant successfully completed a lift at a 10 RPE (maximal effort) with technical proficiency or successfully completed a lift at a lower RPE but failed the subsequent attempt. The squat was performed to International Powerlifting Federation standards which required participants to reach a depth where the hip crease was below the top of the knee joint [29]. The bench press was performed in a touch-and-go style, where participants were required to maintain five points of contact, including their head, shoulders, and buttocks in contact with the bench, and both their feet on the ground for the duration of the lift.

### Training Protocol

Training sessions were supervised, in person when possible, and via video chat when necessary due to COVID-19 restrictions. Training sessions were completed thrice weekly on non-consecutive days at the same time of day whenever possible (occasionally participants rescheduled session times) for eight weeks (23 sessions total). Session layout (Table 3), main lift (Table 4), and accessory lift (Table 5) progressions can be seen in Tables 3, 4 and 5. Warm-up and working sets of squat and bench press were calculated from pre-test 1-RM scores. General main lift progressions maintained three working sets where repetitions per set gradually decreased, while percentage of 1-RM increased throughout most of the program. The first and last week included only two sets as an introductory and tapering stimulus. Additionally, RPE was used to modify RT loads to ensure the percentage of 1-RMs assigned maintained the intended proximity to failure. Accessory exercise loads were determined by the previous week’s training, with the goal of reaching volitional failure within a specified repetition range during each set. Rest periods were set at three minutes between working sets of squats and bench press, and two minutes between accessories, which were performed in circuit fashion (one set per exercise before resting, repeated until all sets were completed).

**Table 3:** Training program

Day 1	Day 2	Day 3
Back Squat	Back Squat	Back Squat
Bench Press	Bench Press	Bench Press
Lat Pulldown	Dumbbell Row	Lat Pulldown
Dumbbell Shoulder Press	Dumbbell Lateral Raise	Dumbbell Shoulder Press
Barbell Curl	Dumbbell Hammer Curl	Barbell Curl

**Table 4:** Squat and bench progression

Week	Day 1	Day 2	Day 3
0—pre-test	×	×	Pre-testing
1—intro	2 × 10 × 60% (5–7 RPE)	2 × 8 × 65% (5–7 RPE)	2 × 6 × 70% (5–7 RPE)
2	3 × 10 × 65% (6–8 RPE)	3 × 8 × 70% (6–8 RPE)	3 × 6 × 75% (6–8 RPE)
3^a^	3 × 10 × 67.5% (6–8 RPE)	3 × 8 × 72.5% (6–8 RPE)	3 × 6 × 77.5% (6–8 RPE)
4	3 × 9 × 70% (7–9 RPE)	3 × 7 × 75% (7–9 RPE)	3 × 5 × 80% (7–9 RPE)
5^a^	3 × 9 × 72.5% (7–9 RPE)	3 × 7 × 77.5% (7–9 RPE)	3 × 5 × 82.5% (7–9 RPE)
6	3 × 8 × 75% (8–10 RPE)	3 × 6 × 80% (8–10 RPE)	3 × 4 × 85% (8–10 RPE)
7^a^	3 × 8 × 77.5% (8–10 RPE)	3 × 6 × 82.5% (8–10 RPE)	3 × 4 × 87.5% (8–10 RPE)
8—taper	2 × 6 × 80% (7–9 RPE)	2 × 4 × 85% (7–9 RPE)	Post-testing

*RPE* rating of perceived exertion based on repetitions in reserve

First set’s load dictated by percentage of pre-test one-repetition maximum. Then adjusted on subsequent sets by RPE; by participant if within range, increased or decreased by 2% for every 0.5 RPE below or above RPE range, respectively

^a^First set’s load is determined by the percentage listed only if initial set was completed on the same day of prior week within or below RPE range. If repetitions were missed or RPE was above range, last week’s load is repeated

**Table 5:** Accessory lift progression (all except back squat and bench press)

Week	Day 1	Day 2	Day 3
0—pre-test	×	×	Pre-testing
1—intro^a^	2 × 10–15	2 × 8–12	2 × 8–12
2	3 × 10–15RM	3 × 8–12RM	3 × 8–12RM
3	3 × 10–15RM	3 × 8–12RM	3 × 8–12RM
4	3 × 10–15RM	3 × 8–12RM	3 × 8–12RM
5	3 × 8–12RM	3 × 6–10RM	3 × 6–10RM
6	3 × 8–12RM	3 × 6–10RM	3 × 6–10RM
7	3 × 8–12RM	3 × 6–0RM	3 × 6–10RM
8—taper^b^	2 × 8–12	2 × 6–10	Post-testing

*RM* repetition maximum

Weeks 2–7: previous week's training consulted to determine load. Goal to fall within repetition range while reaching failure each set. Load increased if more repetitions than target repetition range are completed

^a^Researcher guided load selection with gradual increase from set 1 to set 2 to assess what load can be used in subsequent weeks

^b^Previous weeks loads reduced one increment for machine and dumbbells or 5 kg for barbell lifts

### Statistics

For our group-based comparisons we performed a Bayesian power analysis using the SSDANOVA function in the SSDbain R package [30] using a BF threshold of 3 and power of 0.8. We used baseline muscle thickness variance data from Schoenfeld et al. [31] and baseline skinfold thickness variance data from Ostrowski et al. [32] in the calculations and ran up to 10,000 iterations. We assessed required sample size for a moderate (effect size = 0.5) to large (effect size = 0.8) effect, with the hypothesis that muscle thickness would increase with an increase in calorie intake but no further increase with the highest calorie intake, whereas body fat would increase proportionally with increasing calorie intake. For muscle thickness it was estimated we would need a sample size of 31 or more per group, and for skinfolds we would need 10–22 per group. The statistician was initially blinded to group assignment. Before unblinding, changes in outcomes were analyzed using a Bayesian ANCOVA, with change as the dependent variable, group as the independent variable, and baseline value as a covariate. All models (baseline value, group, group + baseline value) were compared to the null model to produce Bayes factors (BF_10_) which indicate the odds ratio of the likelihood of one hypothesis relative to another where BF_10_ = 1, with values higher than 1 favoring an alternative model and values less than 1 favoring the null model. Strength of evidence in favor of either the null model or alternative models was interpreted according to Andraszewicz et al. [33]. After the ANCOVA, the statistician was unblinded and regressed each outcome for the entire final sample of 17 participants against the change in body mass using Bayesian linear regression. Data were analyzed using JASP 0.16.4 (University of Amsterdam). Data are presented as means ± 95% credible interval (CI)—the 95% probability that the true (unknown) effect estimate lies within the interval, given the evidence provided by the observed data [34] unless otherwise specified.

## Results

### VL_MT_

**Fig. 1:**
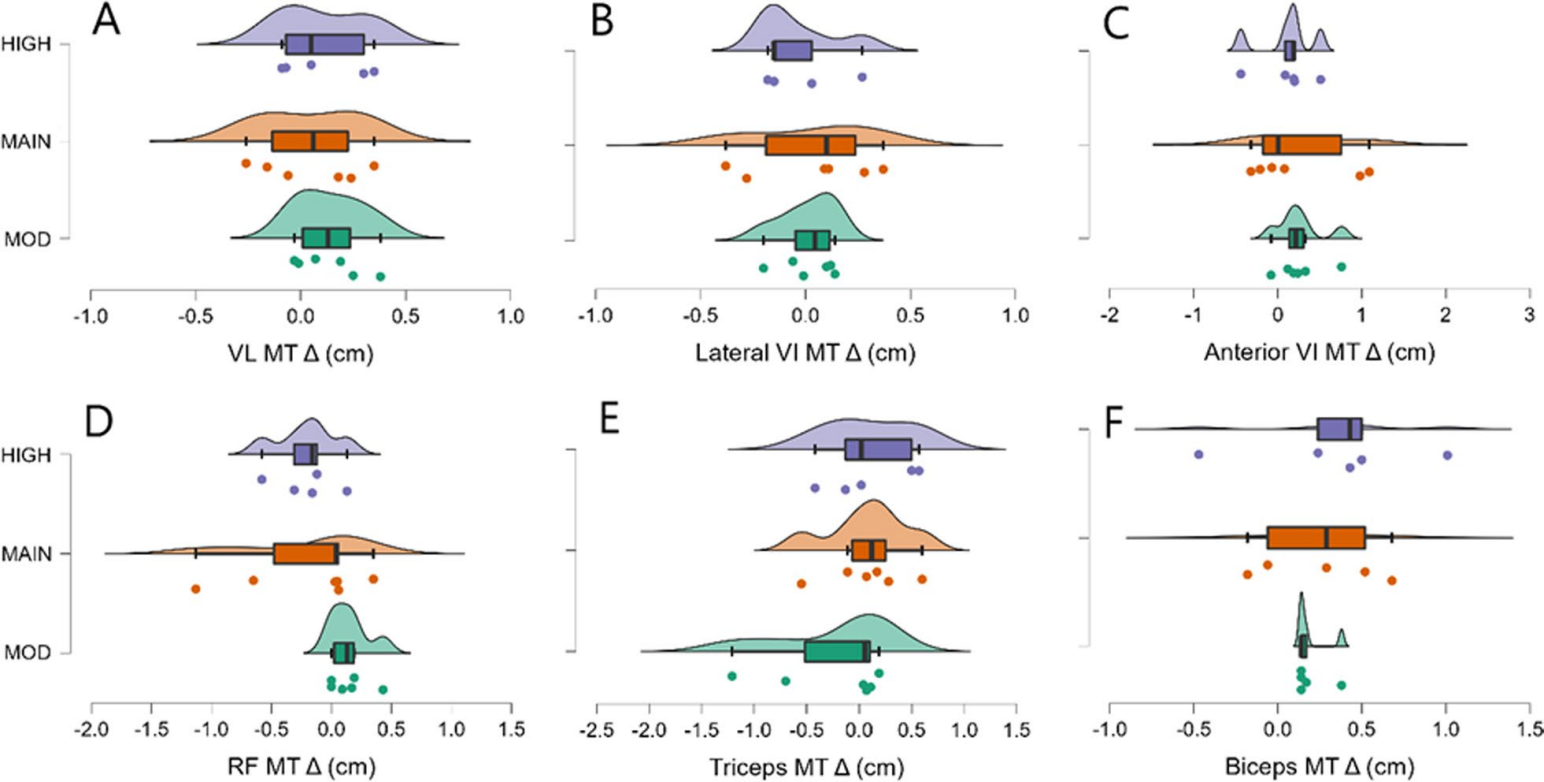
Changes in muscle thickness from pre- to post-intervention. MOD = moderate surplus, MAIN = maintenance, HIGH = high surplus. MT = muscle thickness, Δ = change, VL = vastus lateralis, VI = vastus intermedius, RF = rectus femoris. Box plots illustrate the median, interquartile range, and minimum and maximum values. Individual data points beyond the whiskers are considered outliers (quartile ± 1.5 × interquartile range)

There was moderate evidence in favor of pre-training VL_MT_, but not group, as a predictor of the change in VL_MT_ from pre- to post-assessments (BF_10_ = 3.6, Table 6). There was weak evidence in favor of the null model over group (BF_10_ = 0.35, Table 6). Changes in VL_MT_ were similar among groups (Fig. 1A), and change in body mass was not favored over the null model as a predictor of change in VL_MT_ (BF_10_ = 0.45, *R*^2^ = 0.01).

**Table 6:** Bayesian statistics with Bayes factors (BF) with baseline, group, and baseline and group models for outcome variables

Outcome	Model	BF_10_^a^
VL MT	Pre-training	3.6
	Group + Pre-training	2.2
	Group	0.35
Lateral VI MT	Pre-training	3.3
	Group + Pre-training	1.0
	Group	0.32
Anterior VI MT	Pre-training	1.6
	Group + Pre-training	0.54
	Group	0.33
RF MT	Pre-training	1.5
	Group + Pre-training	1.4
	Group	0.77
Triceps MT	Group	0.52
	Pre-training	0.49
	Group + Pre-training	0.24
Biceps Brachii MT	Pre-training	0.81
	Group + Pre-training	0.37
	Group	0.35
Squat	Pre-training	0.49
	Group	0.31
	Group + Pre-training	0.15
Bench	Group	13.1
	Group + Pre-training	5.9
	Pre-training	0.48
Sum of Skinfolds	Group	3.0
	Group + Pre-training	1.9
	Pre-training	0.65

*BF* Bayes factor, *MT* muscle thickness, *VL* vastus lateralis, *VI* vastus intermedius, *RF* rectus femoris

^a^BF_10_ = 1 indicates an equal likelihood of null and alternate models, with values < 1 favoring the null model and values > 1 favoring the alternate model)

### Lateral VI_MT_

Similar to VL_MT_, there was moderate evidence in favor of pre-training MT, but not group, as a predictor of the change in lateral VI_MT_ (BF_10_ = 3.3, Table 6). There was weak evidence in favor of the null model over the group model (BF_10_ = 0.32, Table 6). Changes in lateral VI_MT_ were similar among groups (Fig. 1B), and change in body mass was not favored over the null model as a predictor of change in lateral VI_MT_ (BF_10_ = 0.43, *R*^2^ = 0.0).

### Anterior VI_MT_

There was weak evidence in favor of pre-training MT, but not group, as a predictor of the change in anterior VI_MT_ (BF_10_ = 1.6, Table 6). There was weak evidence in favor of the null model over the group model (BF_10_ = 0.33, Table 6). Changes in anterior VI_MT_ were similar among groups (Fig. 1C), and change in body mass was not favored over the null model as a predictor of change in anterior VI_MT_ (BF_10_ = 0.51, *R*^2^ = 0.04).

### RF_MT_

There was weak evidence in favor of pre-training MT, but not group, as a predictor of the change in anterior VI_MT_ (BF_10_ = 1.5, Table 6). There was weak evidence in favor of the null model over the group model (BF_10_ = 0.77, Table 6). Changes in RF_MT_ were similar among groups (Fig. 1D), and change in body mass was not favored over the null model as a predictor of change in RF_MT_ (BF_10_ = 0.63, *R*^2^ = 0.08).

### Triceps MT

Evidence weakly favored the null model over all other models (Table 6). Specifically for group, there was weak evidence in favor of the null model over the group model (BF_10_ = 0.52, Table 6). Changes in triceps MT were similar among groups (Fig. 1E), and change in body mass was not favored over the null model as a predictor of change in triceps MT (BF_10_ = 0.43, *R*^2^ = 0.0).

### Biceps MT

Evidence weakly favored the null model over all other models (Table 6). Specifically for group, there was weak evidence in favor of the null model over the group model (BF_10_ = 0.35, Table 6). Changes in biceps MT were similar among groups (Fig. 1F) and change in body mass was only weakly favored over the null model as a predictor of change in biceps MT (BF_10_ = 1.4, *R*^2^ = 0.24).

### Squat 1-RM

**Fig. 2:**
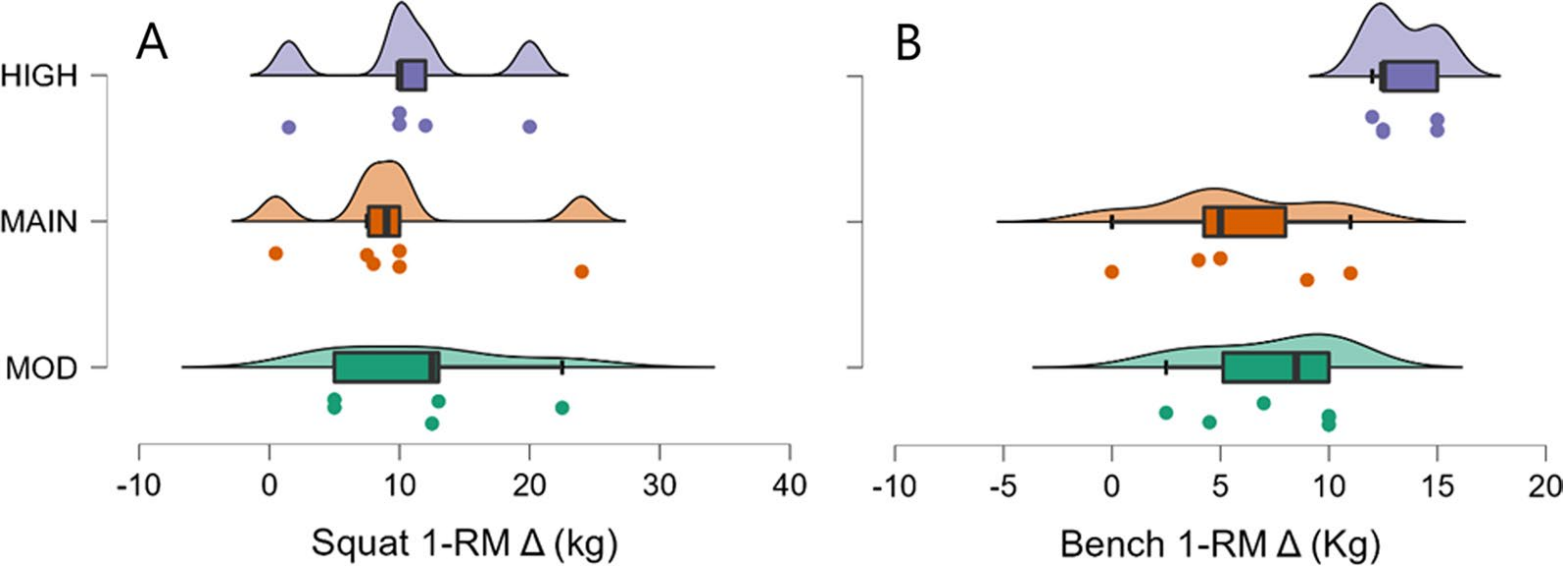
Changes in one-repetition maximum (1-RM) from pre- to post-intervention. MOD = moderate surplus, MAIN = maintenance, HIGH = high surplus. Δ = change. Box plots illustrate the median, interquartile range, and minimum and maximum values. Individual data points beyond the whiskers are considered outliers (quartile ± 1.5 × interquartile range)

Evidence weakly to moderately favored the null model over all other models (Table 6). Specifically for group, there was moderate evidence in favor of the null model over the group model (BF_10_ = 0.31, Table 6). Changes in squat performance were similar among groups (Fig. 2a), and change in body mass was not favored over the null model as a predictor of change in squat performance (BF_10_ = 0.62, *R*^2^ = 0.08).

### Bench 1-RM

There was strong evidence in favor of group over all other models (BF_10_ = 13.1, Table 6). Post hoc comparisons indicated moderate evidence (BF_10_ = 9.9) for group HIGH as having a greater increase compared to group MOD (Fig. 2b), and strong evidence (BF_10_ = 14.5) for group HIGH as having a greater increase compared to group MAIN (Fig. 2b). Change in body mass was not favored over the null model as a predictor of change in bench performance (BF_10_ = 0.97, *R*^2^ = 0.16).

### Sum of Skinfolds

**Fig. 3:**
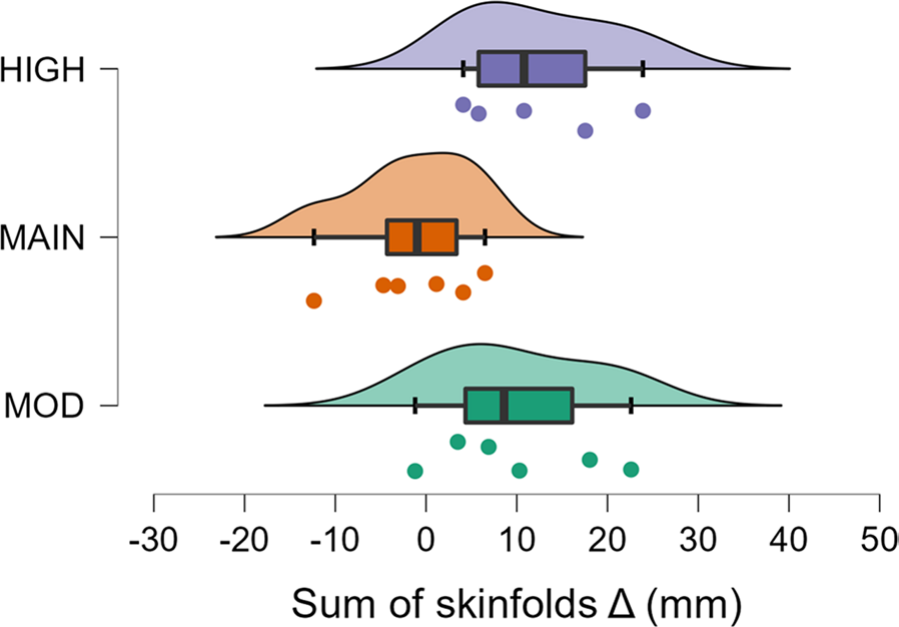
Changes in the sum of skinfolds from pre- to post-intervention. MOD = moderate surplus, MAIN = maintenance, HIGH = high surplus. Δ = change. Box plots illustrate the median, interquartile range, and minimum and maximum values

Evidence moderately favored group over all other models (BF_10_ = 3.0, Table 6). Post hoc comparisons indicated moderate evidence (BF_10_ = 4.2) for group HIGH as having a greater increase as compared to group MAIN (Fig. 3), and weak evidence (BF_10_ = 2.4) for group MOD as having a greater increase as compared to group MAIN (Fig. 3). There was strong evidence that change in body mass was a predictor of change in sum of skinfold thickness over the null model ((BF_10_ = 14.3, *R*^2^ = 0.49).

## Discussion

The present investigation is one of the few existing studies to assess the relationship between the magnitude of energy surplus and change in maximal strength, skinfold thicknesses, and upper and lower body MT in a resistance-trained population. Notably, due to the COVID-19 epidemic and subsequent lockdowns, we were unable to recruit the target *N* before study completion. Therefore, in addition to our a priori planned group-based comparisons, after unblinding we opted to conduct a post hoc regression analysis based on changes in body mass to enhance our statistical power and provide more meaningful conclusions. Our hypotheses linked to our initial group-based statistical comparisons were that both surplus groups would increase MT and 1-RM more than MAIN but similarly to one another, while skinfold increases would scale with the energy surplus size. These hypotheses were partially supported by our findings. Specifically, group-based comparisons for changes in MT weakly favored the null, with similar changes in MT occurring at all sites, in all groups. Similarly, squat 1-RM changes moderately favored the null, with similar increases between groups. However, in partial support of our hypotheses, group-based changes in bench press 1-RM favored the group model with moderate and strong evidence that HIGH outperformed MOD and MAIN, respectively. Finally, the group-based comparisons for changes in the sum of skinfold thicknesses moderately favored the group model, partially in line with our hypotheses. Specifically, there was moderate evidence that HIGH increased their sum of skinfold thicknesses more than MAIN, no evidence of a difference between MOD and HIGH, and only weak evidence than MOD increased their sum of skinfold thicknesses more than MAIN. However, our regression analysis based on body mass changes suggests more of our hypotheses were supported than indicated by our group-based comparisons. Notably, despite no evidence that biceps brachii MT was influenced by group assignment, there was weak support for body mass changes as a predictor of changes in biceps MT. In contrast, despite group-based bench press 1-RM comparisons favoring HIGH, changes in body mass were not favored over the null model as a predictor of bench press 1-RM changes. Lastly, there was strong evidence that changes in body mass predicted changes in the sum of skinfold thicknesses. To summarize, in the context of the studied population following the assigned training protocol, despite 1 RM strength and quadriceps and triceps MT being seemingly unaffected by gains in body mass (and thus, surplus size), participants who gained the most body mass clearly gained the most body fat and (although far less clearly) also gained more biceps MT.

Regarding changes in MT, the only site in which all three groups experienced an arguably meaningful mean increase was the biceps (~ 0.2–0.3 cm). At all other sites, for all groups, mean changes mostly clustered around zero. Given the resistance-trained status of the study population, it is possible that the training protocol provided an insufficient stimulus to produce meaningful quadriceps and triceps hypertrophy for the observed eight-week period. Notably, the only exercise which trained the quadriceps was the squat, for a total of nine sets per week, and while the triceps were trained with 15 sets per week, they were only trained via the multi-joint bench press and shoulder press exercises. However, the biceps were trained for a total of 18 sets per week, with half of these sets coming from multi-joint exercises (row, lat pulldown) and half from isolated elbow flexion (barbell curl and hammer curl). Viewed in light of the most recent meta-analyses on the relationship between weekly set volume and hypertrophy (which also counted combined isolated and multi-joint exercises), it is perhaps unsurprising that biceps MT increased most consistently. Schoenfeld et al. [10] reported a significant (*p* = 0.002) relationship with hypertrophy and weekly muscle-specific set volume in a continuous regression, significantly greater hypertrophy favoring 9 + sets when using a two-category comparison with < 9 sets (*p* = 0.03), and a non-significant (*p* = 0.074) graded dose response using a three-category comparison of 1–4, 5–9, and 10 + weekly sets. Further, in a similar analysis of higher-volume studies, Baz-Valle et al. [35] reported no significant differences between 12–20 and 20 + sets for both biceps and quadriceps, but significantly greater hypertrophy in the triceps when performing 20 + weekly sets compared to 12–20. Given these findings, one would expect the biceps to experience the most hypertrophy of the measured muscles in the present study based on the volumes performed.

Potentially also relevant to our MT findings was the programmed proximity to failure. The bench press and squat exercises were prescribed via %1-RM with the intent to be challenging, yet submaximal. When sets were performed too far from failure (or too close) per the participants perceived repetitions in reserve (RIR) [36], load was adjusted to ensure the successful completion of all repetitions in each set as close to the intended proximity to failure as possible. However, participants were verbally encouraged to train to a 0 RIR on all sets for all other exercises. Indeed, in the most recent meta-analysis [37] on the relationship between proximity to failure and hypertrophy, while the effect size (ES) was trivial to small (0.19), hypertrophy was significantly (*p* = 0.045) greater in groups that trained to failure. Thus, not only were the biceps trained with the highest volume of all measured muscle groups, but they were also trained more intensely. Intriguingly, given that our regression showed weak evidence of a relationship between gains in body mass and biceps MT, it may be that hypertrophy can only be augmented by an energy surplus when the stimulus for a given muscle is sufficiently potent, which may have only been the case for the biceps in the present study. With that said, this supposition should be couched until future confirmatory research is published as the evidence for the relationship between body mass gains and increases in biceps MT was weak (BF_10_ = 1.4, *R*^2^ = 0.24).

All three groups increased squat and bench press 1-RM, and despite the group-based finding that HIGH gained more bench press strength than MOD and MAIN, our regression did not reveal any evidence of a relationship between squat or bench press 1 RM strength gains and increases in body mass. Thus, the training protocol—which was identical between groups—was sufficient to produce maximal strength gains, but an energy surplus (regardless of size) and any subsequent gains in body mass did not augment these gains. Given that, on average, meta-analytic data [12] indicate that even an energy deficit does not significantly impair strength gains (ES = − 0.31, *p* = 0.28)—despite significantly impairing lean mass gains (ES = − 0.57, *p* = 0.02)—our findings that an energy surplus does not augment strength gains are perhaps to be expected. Further, considering there were similar (negligible) increases in triceps and quadriceps MT among groups—the only measured muscles which contribute to squat and bench press performance—one would also not expect to observe any strength differences due to greater contractile tissue gains between groups.

The strongest evidence we observed of an effect related to an energy surplus, was the effect of body mass gains on the increase in the sum of skinfold thicknesses. While our group-based analyses roughly comported with our regression—as both surplus groups increased their sum of skinfolds more than MAIN—they did not align with our hypothesized relationship whereby increases in the sum of skinfolds would follow a pattern of HIGH > MOD > MAIN. Rather, our evidence suggests that both MOD and HIGH, on average, were in a similar energy surplus. This finding specifically highlights a challenge of conducting ecologically valid translational research, as the intended difference between groups did not occur despite regular contact between participants and a skilled researcher with clinical nutrition experience. It is possible that this finding also indicates that a prescribed 5% surplus is too small to be logistically feasible for most participants, on average, and in practice becomes a larger surplus. Nonetheless, our regression sidesteps this challenge, highlighting the clearer, strong relationship between body mass gains (and thus, the individual energy surplus magnitude) and sum of skinfold thickness changes. Furthermore, given the expected variability in body mass gains when assigning participants energy surplus values in applied settings [16], researchers conducting similar future studies should consider an a priori continuous analyses rather than a group-based design. To summarize, it seems the clearest and strongest impact of a larger energy surplus is an increase in body fat, at least in the context of the present RT protocol and study population. Given the aforementioned weak relationship between body mass increases and biceps MT increases, it is possible that had a more potent RT stimuli for all muscles been imposed, such a program could have mitigated these gains in body fat to some degree (as more of the energy surplus might have been partitioned toward lean tissue accrual). On this note, for study feasibility, a 3-day per week full-body training protocol was implemented. This approach is in contrast with the typical 4–6-day body part split-routines implemented by most bodybuilders [38] which based on a meta-analysis of training frequency may be more conducive to completing higher training volumes [39]. However, without further study it is difficult to confirm whether or not a higher volume, 4–6-day routine would allow slightly greater muscle gain in a larger surplus, or whether the trade-off between greater increases in body fat would be worth the likely proportionally smaller increases in muscle mass (based on the stronger relationship between body mass gains and increases in skinfolds rather than biceps thickness). In practice, the value of this trade-off might depend on the context of the individual. For example, someone with body aesthetic goals might choose slower weight gain to mitigate gains in body fat, while an American football lineman who benefits not only from increases in muscle mass, but also increases in body mass (to some degree regardless of composition), may choose faster weight gain.

Ours is one of the few studies on the effect of variable energy surpluses among resistance-trained participants. Previously, Garthe and colleagues [17] conducted an individualized 8–12-week nutritional intervention in 39 resistance-training elite athletes designed to enhance muscle gain. Specifically, participants were divided between two groups, one group was guided by a dietitian to reach a specific, modest daily energy surplus, while participants in the comparative group followed a self-guided nutritional approach. The dietitian intervention led to the athletes consuming 3585 ± 601 kcal/d; ~ 600 kcal greater than the comparative group. In line with our findings, despite a fivefold greater increase in fat mass in the dietitian-guided group (15 ± 4 vs. 3 ± 3%) there were no significant differences between groups in strength or lean body mass increases. In somewhat of a contrast, using a Bayesian modeling approach, Smith and colleagues [16] reported that gains in body mass were a significant predictor of fat-free mass increases in a group of 21 resistance-trained (minimum 6-month experience) adults during a concurrent overfeeding and resistance-training protocol. For six weeks participants consumed a high energy protein and carbohydrate supplement with the goal of gaining 0.45 kg/wk—although actual changes in body mass varied between participants—while performing three supervised weekly RT sessions. Despite a great deal of interindividual variability, the authors’ model predicted that a body mass gain of ~ 0.55%/week was indicative of all body mass gains being fat-free mass (*R*^2^ = 0.36). In an even more stark contrast to both the present and aforementioned findings, Rozenek and colleagues [40] reported nearly exclusive lean mass gains in a group of untrained subjects following a relatively large energy surplus (~ 3-kg body mass increase in eight weeks) while RT. Ultimately, larger surpluses are likely to cause excess gains in body fat, but the degree to which relatively larger or smaller surpluses impact gains in muscle mass is variable between individuals, and possibly impacted by the quality of the training program, its appropriateness for a given study group, which may be at least in part based on their prior training experience and history, with less-trained participants more capable of benefitting from larger surpluses.

The limitations of the present study are notable. Drops outs and delays due to COVID-19 resulted in an *N* roughly 60% of what we intended, and thus, our group-based comparisons might be inaccurate due to an insufficient sample size. Further, group assignment was based on the target energy surplus; however, despite competent and consistent monitoring, the intended energy surpluses were not consistently followed by all participants. However, we sought to mitigate these limitations by performing a post hoc regression analysis on body mass as a continuous variable which both strengthens our sample size and corrects these energy surplus discrepancies. In addition to these limitations, despite our best intentions, we also had a primarily male sample (two out of 17 participants were female). Thus, future research is required to elucidate any potential sex differences. Additionally, given the relatively large variances in changes in skinfold and MT, the individual responses to the nutritional and RT interventions suggest that a larger sample size may provide a clearer answer on population-level responses. Finally, as mentioned, a different, more potent training protocol could have produced different results. While “resistance-trained” participants were recruited, our participants were diverse in strength, training experience, and goals. While all met the requirements for participation, some were well above them. There were several competitive strength and physique athletes in our sample alongside those who consider themselves recreational lifters. In many cases our participants habitually trained with more frequency, and in some cases more volume than they performed during this investigation. Thus, logistics permitting, in future study researchers should endeavor to conduct a “lead in” training period where all participants follow the same low- to moderate-volume protocol in attempt to homogenize individual differences in training status before starting the actual study protocol. Further, in well-trained overfeeding populations, higher-frequency and volume protocols may prove produce more favorable body composition changes than we observed. If more directly supervised training sessions are not feasible, we recommend perhaps including 1–2 self-guided sessions (or sessions supervised by video) in addition to the typically conducted 2–3 supervised, laboratory-based training sessions.

## Conclusions

In the present sample, individuals who consumed larger energy surpluses—thereby gaining more body mass—experienced similar increases in strength and triceps and quadriceps muscle size but increased their skinfold thicknesses more compared to those who consumed smaller energy surpluses or maintenance calories. There was, however, weak evidence that larger surpluses resulted in greater gains in biceps thickness; the muscle group trained with the most volume and intensity. Thus, if an overfeeding strategy is followed, it may be more successful from a body composition standpoint if accompanied by a more stimulative training protocol for all muscle groups. Ultimately, however, given clearer evidence and a much stronger relationship between body mass gains and increases in the sum of skinfold thicknesses, we recommend conservative energy surpluses scaled to RT experience of 5–20% over maintenance energy or rates of weight gain of 0.25–0.5% of body mass per week, scaled to RT experience such that more advanced trainees consume smaller surpluses and gain weight more slowly [3].

## Data Availability

The datasets used and/or analyzed during the current study are available from the corresponding author on reasonable request.

## Abbreviations

RT: Resistance training
HIGH: 15% Energy surplus with training group
MOD: 5% Energy surplus with training group
MAIN: Maintenance energy with training group
MT: Muscle thickness
1-RM: One-repetition maximum
VL: Vastus lateralis
VI: Vastus intermedius
RF: Rectus femoris
RPE: Rate of perceived exertion
CI: Credible interval
RIR: Repetitions in reserve
ES: Effect size
